# GBP1 Facilitates Indoleamine 2,3-Dioxygenase Extracellular Secretion to Promote the Malignant Progression of Lung Cancer

**DOI:** 10.3389/fimmu.2020.622467

**Published:** 2021-01-20

**Authors:** Yinnan Meng, Wei Wang, Meng Chen, Kuifei Chen, Xinhang Xia, Suna Zhou, Haihua Yang

**Affiliations:** ^1^ Laboratory of Cellular and Molecular Radiation Oncology, Radiation Oncology Institute of Enze Medical Health Academy, Department of Radiation Oncology, Affiliated Taizhou Hospital of Wenzhou Medical University, Taizhou, China; ^2^ School of Medicine, Shaoxing University, Shaoxing, China

**Keywords:** lung cancer, indoleamine 2,3-dioxygenase 1 (IDO1), GBP1, PD-1, astragaloside IV

## Abstract

IDO1-mediated immune escape can lead to the malignant progression of tumors. However, the precise mechanism of IDO1 remains unclear. This study showed that IDO1 can bind to GBP1 and increase the extracellular secretion of IDO1 with the assistance of GBP1, thereby promoting the malignant proliferation and metastasis of lung cancer. In vitro study showed that the high expression levels of IDO1 and GBP1 in lung cancer cells promoted cell invasion and migration. In vivo study revealed that knock-down of IDO1 and GBP1 inhibited tumor growth and metastasis. In addition, Astragaloside IV reduces the extracellular secretion of IDO1 by blocking the interaction of IDO1 and GBP1, thereby reducing T cell exhaustion and inhibiting tumor progression. These results suggest that blocking the extracellular secretion of IDO1 may prevent T cell exhaustion and thereby enhance the effect of PD-1 inhibitors on cancer treatment.

## Introduction

Lung cancer is the leading cause of cancer deaths in the world. Non-small cell lung cancer accounts for 80% of all lung cancers. It is usually diagnosed in the advanced stage, and the 5-year survival rate is very low (1, 2). Non-small cell lung cancer includes squamous cell carcinoma, adenocarcinoma and large cell carcinoma. Compared with that of other subtypes of lung cancer, the incidence of lung adenocarcinoma is relatively low, but the prevalence has increased, and blood metastases often occur early (3, 4). Surgery remains the standard method to treat lung cancer. Chemotherapy drugs and targeted drugs for EGFR mutation, BRAF mutation and ALK translocation are also used in the treatment of lung cancer. In addition, the development of programmed cell death 1 (PD-1)/PD-1 ligand 1 (PD-L1) checkpoint inhibitors has changed the pattern of non-small cell lung cancer treatment and achieved certain therapeutic effects (5).

Indoleamine 2,3-dioxygenase (IDO) is a home-containing enzyme that is highly expressed in myeloid cells and catalyzes the initial step of tryptophan degradation. Tryptophan starvation caused by IDO inhibits T cell function through several mechanisms (6, 7). An increasing number of studies linked the overexpression of IDO with cancer progression. High levels of IDO have been found in patients with ovarian cancer, hepatocellular carcinoma, invasive cervical cancer, non-small cell lung cancer, and are associated with poor prognosis (8–12). Although some studies support that blocking IDO exerts a positive anti-tumor effect, the mechanism of how to regulate IDO in tumor cells remains unclear.

This study showed that IDO1 can bind to GBP1. With the assistance of GBP1, the extracellular secretion of IDO1 increased. In vivo and *in vitro* experiments showed that the simultaneous overexpression of IDO1 and GBP1 can promote the migration and invasion of lung cancer cells. In addition, astragaloside IV can block the combination of IDO1 and GBP1 to inhibit the progression of lung cancer. These results provide a certain theoretical basis for the combined targeted therapy of IDO1 and PD-1 and may provide a new idea for the clinical treatment of lung cancer.

## Materials and Methods

### Cell Culture

Lung cancer cell lines NCI-H460, A549 were purchased from cell resource center of Chinese academy of medical sciences, and NCI-H345, NCI-H1299, NCI-H146, NCI-H1341 cells were purchased from ATCC. Mouse Lewis Lung Cancer cell LLT was obtained from cell resource center of Chinese academy of medical sciences. NCI-H345, NCI-H1299, NCI-H146, NCI-H1341 cells were purchased from ATCC. Cells were maintained in RPMI-1640 (Gibco, USA) or DMEM (Gibco, USA) medium supplement with 10% FBS (Gibco, USA). All cells were cultured at 37°C under a humidified atmosphere of 5% CO2.

### Silver Staining

At 48 h after cell transfection, cell extracts were obtained using lysis buffer (0.2 mM EDTA, 50 mM Tris-HCl, 150 mM NaCl and 0.3% NP-40). The anti-Flag tag affinity beads (Sigma, USA) were incubated with cell extracts at 4°C overnight. After washing the beads with a cold lysis buffer containing 0.1% NP-40, Flag peptide was applied to the beads to elute the Flag protein complex. The obtained protein was separated *via* 10% SDS-PAGE and then identified using a silver staining kit (Beyotime, China). After the electrophoresis, the gel was placed in the fixative for about 1 h, washed with 30% ethanol for 10 min and then washed with 200 ml of pure water for 10 min. Then, silver solution was added for 10 min and then rinsed with ice and water until color developed. Finally, a stop solution was added to stop the reaction and obtain different bands.

### Colony Formation Assay

After reaching the logarithmic growth phase, the transfected cells were digested with 0.25% trypsin and gently pipetted to make them single cells. After the cell count, the cells were seeded into a six-well plate at a density of 1000 cells per dish and placed in a 37°C 5% CO2 incubator for 15 days. When macroscopically visible clones were present, the supernatant was discarded, and the cells were washed twice with PBS. The cells were fixed with 4% paraformaldehyde for 15 min and then stained with GIMSA staining solution for 20 min. After washing with running water and drying, the number of colonies that formed was counted.

### Transwell

Cells with a 1:6 dilution of 50 mg/L Matrigel (BD, USA) were inoculated with a final concentration of 1 × 10^5^ cells per well and then cultured in top chamber (Corning, USA) with serum-free medium (Gibco, USA). The complete medium containing 10% FBS was added to bottom and then cultured for 16 h. Then, the cells were removed, and those cells that have not passed were wiped off with a cotton swab. After fixing with formaldehyde for 30 min at room temperature, the cells were stained with 0.1% crystal violet for 20 min. After washing three times with clean water, the cells were observed under a microscope (Nikon, Japan) and then counted.

## Wound Healing

The cells were seeded into six-well plates. When the cell confluence reached 90% after transfection, a straight scratch was made with a 100-μl pipette tip. The cells were washed with PBS three times to remove the suspended cells, added with medium and then placed in a 37°C, 5% CO2 incubator. After 24 h, photographs of the cells were taken, and then the cell migration rate was calculated.

### Plasmid and Lentivirus

Wild-type GBP1 and IDO1 overexpression plasmids were purchased from Sinobiogical (Beijing, China). The mutant IDO1 plasmid was synthesized by Genwiz (Suzhou, China) and inserted into the pCMV3-N-FLAG vector and was verified by DNA sequencing. The synthesized NRP1 and IDO1 shRNA sequences were annealed and inserted into the pLKD-CMV-Puro-U6-shRNA vector, and finally packaged into lentivirus in 293FT cells. shRNA sequences were as follows: GBP1-top: 5′-CACCGCCTCATTGAGAACACTAATGCGAACATTAGTGTTCTCAATGAGGC-3′, GBP1-bot: 5′-AAAAGCCTCATTGAGAACACTAATGTTCGCATTAGTGTTCTCAATGAGGC-3′, IDO1-top: 5′-CACCGCCAAGAAATATTGCTGTTCCCGAAGGAACAGCAATATTTCTTGGC-3′, IDO1-bot: 5′-AAAAGCCAAGAAATATTGCTGTTCCTTCGGGAACAGCAATATTTCTTGGC-3′. All plasmid transfections were performed using Lipofectamine 3000 in accordance with the instructions. A lentivirus was constructed and used in the *in vivo* experiments.

### Molecular Docking

The GBP1 and IDO1 protein crystal structures were downloaded from the PDB database (http://www.rcsb.org). HEX software was used for molecular docking, and the binding force was analyzed based on protein surface potential and surface structure. On the basis of the docking results, the protein interaction interface was used as the active pocket for the high-throughput screening of inhibitors.

### Western Blot

After discarding the medium and washing with 1× PBS, the cells were treated with PMSF-containing lysis solution (50 mM Tris, 150 mM NaCl, 1% Triton X-100, 1%sodium deoxycholate, 0.1% SDS) and incubated for 30 min. For the detection of the IDO1 protein level in the medium, we collected 60 ml of the medium and centrifuged at 3,000*g* for 20 min using a 30-kDa protein concentrator (Millipore, USA) to obtain a protein mixture. The total protein obtained was harvested, quantified by BCA method, separated through SDS-PAGE and then transferred onto the PVDF membrane (Millipore, USA). After blocking with 5% BSA for 2 h, the membrane was incubated with primary antibodies for 4 h at room temperature and then with HRP-labeled secondary antibodies for 2 h at room temperature. Finally, the band was visualized using an enhanced chemiluminescence system (Millipore, USA) in accordance with the manufacturer’s instructions. Primary antibodies were as followed: IDO1 (Abcam, USA, ab134197, 1:1000), GBP1 (Proteintech, China, 67161-1-Ig, 1:500). GAPDH (Abcam, USA, ab8245, 1:3000). Each experiment was repeated three times.

### Immunofluorescence

The treated cells were seeded on a glass slide in a 24-well plate and cultured at 37 ℃ and 5% CO2 for 48 h. After immersion in 1× PBS for three times, the cells were fixed with 4% paraformaldehyde (Beyotime, China) for 15 min. Then, the cells were treated with 0.5% Triton X-100 at room temperature for 20 min, washed with 1× PBS, added with normal goat serum (Solarbio, Beijing, China) and blocked for 30 min at room temperature to remove non-specific binding. The slides were incubated with the following primary antibodies at 4°C overnight. GBP1 (Proteintech, China, 67161-1-Ig, 1:500), IDO1 (Abcam, USA, ab134197, 1:1000). After washing with PBST for three times, the cells were added with the diluted fluorescent-labeled secondary antibody (Santa Cruz, USA), incubated at 37°C for 1 h and finally incubated with DAPI for 5 min in the dark. The collected images were observed under a fluorescence microscope (Nikon, Japan).

### Xenograft Model

Thirty-two 6-week-old Balb/c nude mice were randomly divided into four groups. NCI-H1299 cells were inoculated subcutaneously at a concentration of 5×10^6^ cells per mouse. When the diameter of the tumor reaches 5 mm, GBP1 and IDO1 knock-down lentivirus were used for intertumoral injection. For the metastasis model, luciferase-labeled NCI-H1299 cells were injected into the tail vein to establish a tumor metastasis model. Ten days later, GBP1 and IDO1 knock-down lentivirus were used for intertumoral injection. In order to test the synergistic effect of astragaloside IV and PD-1 inhibitors, C57 mice inoculated with Lewis lung cancer cells. 9 days after inoculation, the tumor diameter reaches 5 to 7 mm, mice were injected with either astragaloside IV (5 mg/kg), anti-PD-1 antibody (BioXCell, 200 μg/mouse, intraperitoneally administered) or the combination of both agents. The tumor volume and animal survival status were monitored every 3 days. Thirty days after cell inoculation, the animals were euthanized by intravenous injection of pentobarbital sodium at the final concentration of 100 mg/kg. The solid tumors were harvested and fixed with formalin for immunohistochemical detection. The tumor volume was calculated as follows: V(volume)=(length × width^2^)/2. All animal experiments were conducted in accordance with the ethical standards of the Institutional Animal Care and Use Committee of the Affiliated Taizhou hospital of Wenzhou Medical University.

### Flow Cytometry

Solid tumors were digested in collagenase and DNase I and dissociated according to the instructions of tumor dissociation kit (Miltenyi Biotec, Auburn, CA). Tumor-infiltrating lymphocyte were enriched and harvested separately by Percoll gradient (Sigma, USA). The lymphocytes were then washed and subsequently blocked with Fc antibody (BD Pharmingen) for 10 min on ice followed by incubation or staining with cell surface antibodies: CD3-APC (Biolegend, USA), CD8-PE (Biolegend, USA) for a 30-min incubation on ice. Cells were washed with Fix/Perm buffer and assayed on a guava easyCyte flow cytometer (MILLIPORE, USA).

### Immunohistochemistry

After deparaffinization of 4-μm paraffin sections, 0.3% H_2_O_2_ was added to inhibit endogenous peroxidase activity. The slices were placed in 0.01 M sodium citrate buffer solution (pH 6.0) and then heated in a microwave oven for antigen retrieval. Subsequently, the slices were added with 5% negative serum to block non-specific proteins and with the corresponding primary antibodies and then incubated overnight at 4°C. After discarding the primary antibodies and washing with 1× PBS, the slices were added with the diluted secondary antibody and incubated at 37°C for 30 min. Finally, DAB was added for color visualization, and the staining intensity was recorded under a microscope.

### Statistical Analysis

All statistical analyses were performed using SPSS 19.0 (SPSS, Chicago, USA). The results are expressed as mean ± standard deviation (SD). Student’s t test was used to compare the significant differences between the two groups. Comparisons between three or more groups were performed using ANOVA and Dunnett’s post-test. Statistical significance was considered at P < 0.05 and marked with *.

## Results

### Indoleamine 2,3-dioxygenase 1 Interacts With GBP1 in Lung Cancer Cells

**Figure 1:**
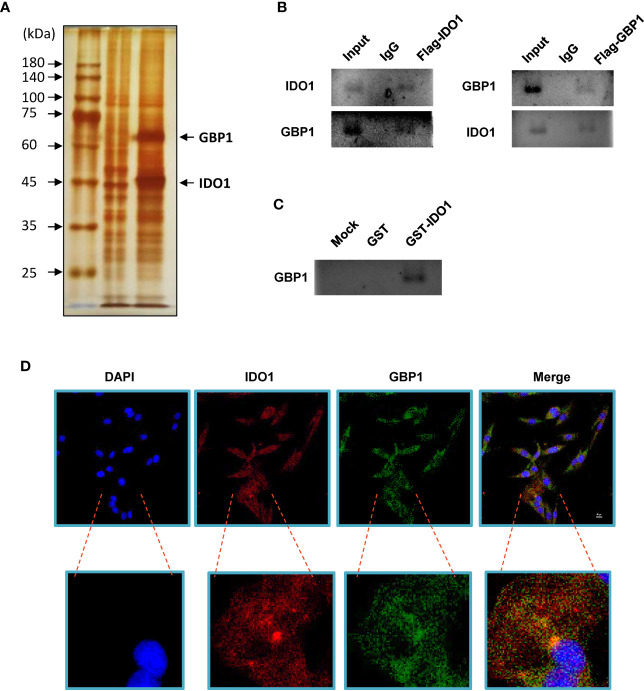
IDO1 binds GBP1 in A549 cells. **(A)** Identification of IDO1 binding protein by silver staining. **(B) ** Use Co-IP to detect the interaction between IDO1 and GBP1. **(C)** GST pull-down was performed to analyze the interaction between IDO1 and GBP1 in vitro. **(D)**. Immunofluorescence confirms the co-localization of IDO1 and GBP1 in A549 cells.

Flag-tagged IDO1 overexpression plasmid was transfected into A549 cells. Anti-flag magnetic beads were used to obtain the IDO1 binding protein complex and separated by PAGE gel. After silver staining, it was found that there was an obvious band near 65 kDa, which was identified as GBP1 by mass spectrometry (**Figure 1A**). In order to confirm the interaction between IDO1 and GBP1 *in vivo*, Co-immunoprecipitation was performed. Results showed that GBP1 signal could be detected in the protein complex obtained with anti-flag magnetic beads after transfection of Flag-IDO1. Similarly, GBP1 could also capture IDO1 in cell lysates (**Figure 1B**). GST pull-down experiment was used to detect the interaction between IDO1 and GBP1 *in vitro*. It was found that GST fused IDO1 and transcribed GBP1 *in vitro* can indeed bind to each other (**Figure 1C**). In addition, we used immunofluorescence to determine the co-localization of IDO1 and GBP1 in A549 cell species. The results showed that IDO1 and GBP1 had colocalization signals (**Figure 1D**).

### Indoleamine 2,3-dioxygenase 1 and GBP1 Promotes Lung Cancer Cell Migration and Invasion

**Figure 2:**
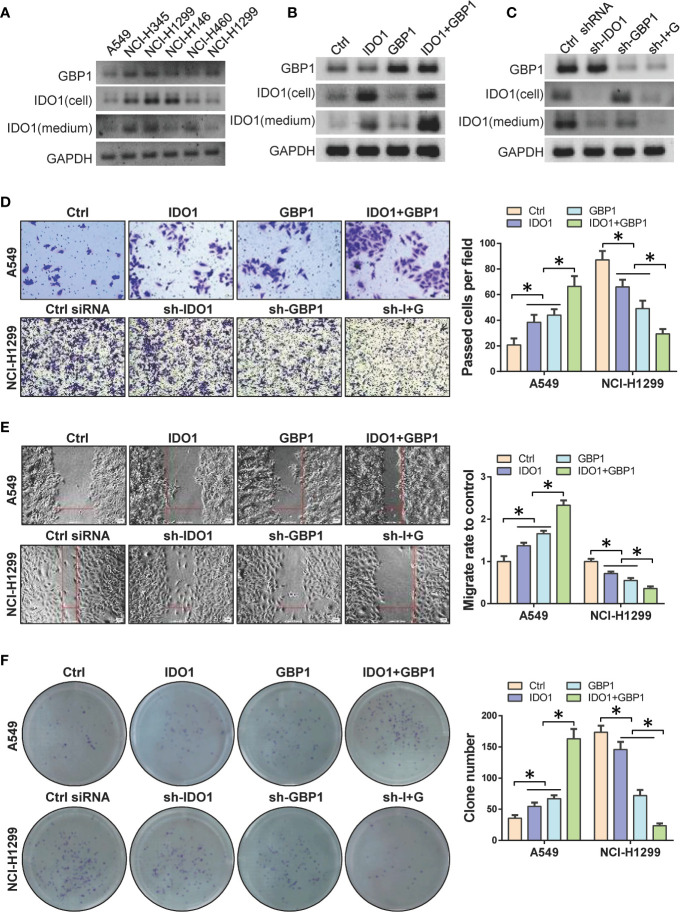
DO1 and GBP1 promotes lung cancer cell invasion, migration and colony formation ability. **(A)** Western blot to detect the protein of IDO1 and GBP1 in 6 lung cancer cell lines. **(B)** Protein levels of GBP1 and IDO1 in cell and culture medium after ectopic expression of IDO1 and GBP1. **(C)** Protein levels of GBP1 and IDO1 in cell and culture medium after knocking down of IDO1 and GBP1. **(D)** Transwell to analyze cell invasion ability after GBP1 and IDO1 overexpression. **(E)** Wound healing to analyze cell migration ability after GBP1 and IDO1 ectopic expression. **(F)** Detection of cell colony formation ability. Statistical significance was considered at P < 0.05 and marked with *.

In order to verify the role of IDO1 and GBP1 in lung cancer species, western blot was performed to detect the expression levels of IDO1 and GBP1 in 6 lung cancer cell lines. The results showed that the protein levels of IDO1 and GBP1 were the highest in NCI-H1299 cells and the lowest in A549 cells (**Figure 2A**). Therefore, we selectively overexpressed IDO1 and GBP1 in A549 cells (**Figure 2B**) and knock down IDO1 and GBP1 in NCI-H1299 cells (**Figure 2C**). We found that GBP1 contributed to the extracellular secretion of IDO1. Then the effects of IDO1 and GBP1 on the invasion, migration and colony formation ability of lung cancer cells were tested by transwell (**Figure 2D**), wound healing (**Figure 2E**) and colony formation (**Figure 2F**) assay. Results showed that overexpression of IDO1 or GBP1 promoted the invasion, migration and colony formation ability of A549 cells compared that of control vector group. Compared with the overexpression of IDO1 or GBP1 respectively, co-overexpression of IDO1 or GBP1 promoted the cells more obviously. In NCI-H1299 cells, knocking down IDO1 or GBP1 could inhibit cell invasion, migration. These experiments indicated that IDO1 and GBP1 contributed to the malignant progression of lung cancer, which may due to the interaction between IDO1 and GBP1.

### GBP1 Contributes to the Extracellular Secretion of Indoleamine 2,3-dioxygenase 1

**Figure 3:**
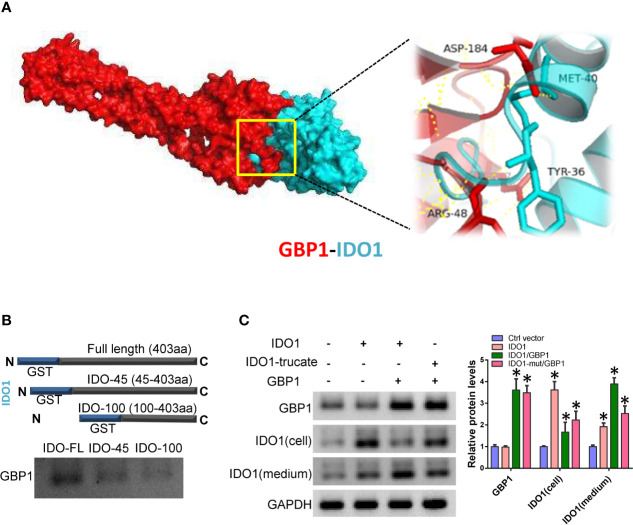
GBP1 contributes to IDO1 extracellular secretion. **(A)** Molecular docking simulates the binding between IDO1 and GBP1. **(B)** Interaction of truncated IDO1 and GBP1 *in vitro*. **(C)** Expression of GBP1 and IDO1 in cells and culture media. Statistical significance was considered at P < 0.05 and marked with *.

In order to verify the precise mechanism of GBP1 and IDO1 interaction and its role in lung cancer cells, we simulated the interaction between GBP1 and IDO1 using molecular docking. The results showed that IDO1 protein have strong binding capacity to GBP1 (**Figure 3A**). Next, we performed in GST pull-down between truncated GST-fused IDO1 proteins and *in vitro* transcribed GBP1 proteins. The results showed that after removing the 45 amino acids at the N-terminus, the binding ability of the truncated IDO1 and GBP1 was weakened (**Figure 3B**). In addition, we co-express wild-type or mutant IDO1 with GBP1 in A549 cells. The results showed that GBP1 could assist the secretion of wild-type IDO1 out of the cell. However, after IDO1 mutation, GBP1’s carrying effect is weakened (**Figure 3C**). These results suggest that the interaction with GBP1 may contribute to extracellular localization of IDO1 is closely related to GBP1.

### Indoleamine 2,3-dioxygenase 1 and GBP1 Promotes Tumor Genesis and Metastasis *In Vivo*

**Figure 4:**
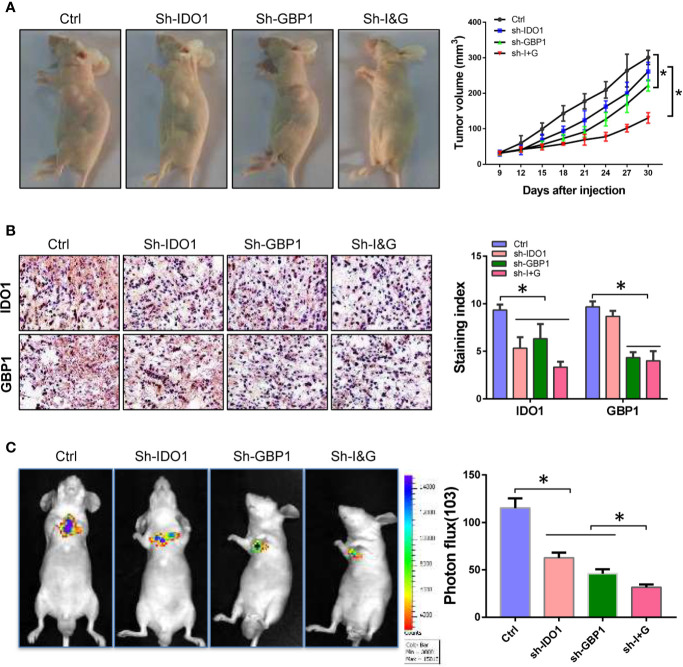
Knock-down of IDO1 and GBP1 inhibits lung cancer tumorigenesis and metastasis. **(A)** Pictures of tumor-bearing mice and tumor volume. **(B)** The expression and subcellular location of IDO1 and GBP1 detected by immunohistochemistry. **(C)** Images of lung metastases and relative fluorescence intensity of metastatic tumor. Statistical significance was considered at P < 0.05 and marked with *.

The above results suggested that GBP1 can promote the extracellular secretion of IDO1, which may be involved in the malignant progression of lung cancer *in vitro*. Here we inoculated NCI-H1299 cells into Balb/c nude mice by subcutaneous inoculation and tail vein injection at a concentration of 10^6^ cells per mice. Seven days after inoculation, sh-IDO1 and sh-GBP1 lentiviruses were injected into tumors individually or jointly every 5 days. For tail vein inoculated animals, after 7 days of inoculation, sh-IDO1 and sh-GBP1 lentiviruses were injected through the tail vein alone or jointly transfected with two retroviruses every 5 days. After 35 days, all the mice were euthanized by carbon dioxide asphyxiation. Then tumor volume was measured, and the tumor tissue was formalin fixed and used for immunohistochemical staining to detect the protein expression of IDO1 and GBP1. The results showed that after IDO1 and GBP1 were knocked down, tumor volumes were decreased, and the tumor volume was the smallest in the IDO1 and GBP1 group (**Figure 4A**). The results of immunohistochemistry showed that knocking down IDO1 and GBP1 can reduce the extracellular secretion of IDO1, while knocking down IDO1 and GBP1 simultaneously prevented the extracellular secretion of IDO1 to a greater extent (**Figure 4B**). In tail vein mice, knocking down IDO1 and GBP1 can inhibit the metastasis of lung cancer cells (**Figure 4C**).

### Astragaloside IV Blocks Indoleamine 2,3-dioxygenase 1 and GBP1 Interaction and Inhibits Lung Cancer Cell Progression

**Figure 5:**
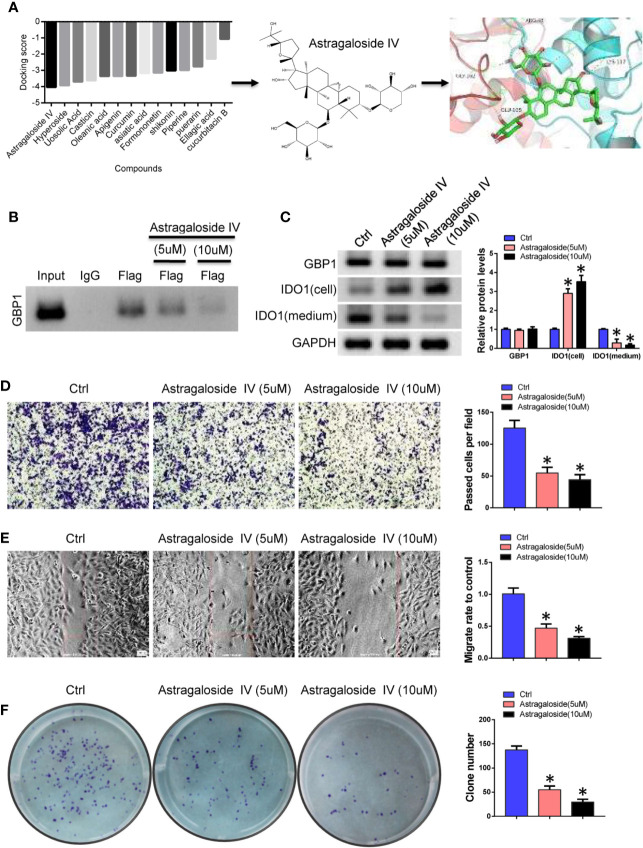
Astragaloside IV blocks the interaction of IDO1 and GBP1. **(A)**. Screening of interface inhibitors and the binding of astragaloside IV to IDO1 and GBP1 interaction regions. **(B)** Co-IP was performed to detect the effect of astragaloside IV between IDO1 and GBP1 binding. **(C)** Western blot detects the expression of GBP1 and IDO1. **(D)** Transwell to evaluate the role of astragaloside IV on cell invasion. **(E)** Cell migration was detected by wound healing assay. **(F)** The role of astragaloside IV on cell colony formation ability. Statistical significance was considered at P < 0.05 and marked with *.

Based on the above results, we speculate that GBP1-mediated extracellular secretion of IDO1 enhances the malignancy of tumor cells. According to the results of molecular docking, the interaction region of GBP1 and IDO1 was used as the active pocket for screening of interface inhibitors. The results showed that astragaloside IV has a stronger binding force in the interaction area and may block the combination of IDO1 and GBP1 (**Figure 5A**). Considering that the existing studies have proved the anti-tumor effect of astragaloside IV, we selected astragaloside IV as a candidate interface inhibitor for IDO1 and GBP1. After treating NCI-H1299 cells with 5 and 10 µM astragaloside IV, the binding ability of the truncated IDO1 and GBP1 was weakened (**Figure 5B**). To detect the protein expression of GBP1 and IDO1, we found that astragaloside IV can prevent the extracellular localization of IDO1 (**Figure 5C**). Next, we use transwell, wound healing and colony formation assays to analyze the effects of astragaloside IV on the invasion, migration of lung cancer cells. The results show that compared with control group, Astragaloside IV (5 and 10 μM) can inhibit cell invasion (**Figure 5D**), migration (**Figure 5E**) and colony formation ability (**Figure 5F**). This indicated that inhibiting the extracellular secretion of IDO1 by blocking the interaction of IDO1 and GBP1 can also inhibit the malignant progression of lung cancer cells.

### Astragaloside IV Inhibits Tumor Genesis and Metastasis *In Vivo*

**Figure 6:**
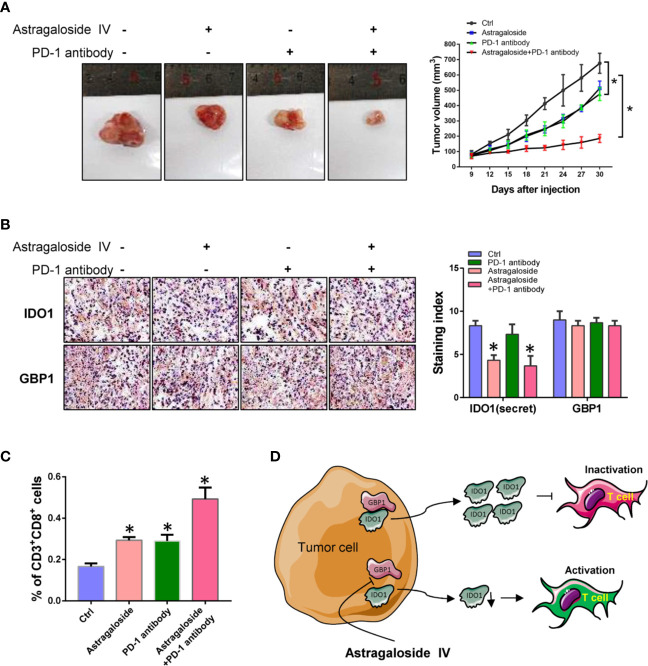
Astragaloside IV combined with PD-1 inhibitor represses tumor growth of lung cancer. **(A)**. The effect of astragaloside IV combined with PD-1 inhibitors on tumor growth. **(B)**. Detection of IDO1 and GBP1 expression by immunohistochemistry. **(C)**. Percentage of tumor infiltrating CD3^+^CD8^+^ T cells. **(D)**. Schematic diagram of this study. Statistical significance was considered at P < 0.05 and marked with *.

Because IDO1 can inhibit the anti-tumor effect of T cells by degrading tryptophan. Therefore, blocking the extracellular secretion of IDO1 may enhance the anti-tumor effect of PD-1 inhibitors. Here we inoculate Lewis lung cancer cells into C57 mice by subcutaneous injection. After combined treatment with astragaloside IV and PD-1 antibody, the effect on tumor growth was evaluated. The results showed that astragaloside IV can enhance the inhibitory effect of PD-1 inhibitors on tumor growth (**Figure 6A**). By using IHC, it was found that astragaloside IV had no effect on the expression of GBP1, but the amount of extracellular secretion of IDO1 was reduced (**Figure 6B**). This can further confirm that astragaloside IV blocks the extracellular localization of IDO1 to enhance the anti-tumor effect of PD-1 inhibitors. In addition, the use of flow cytometry analysis found that the proportion of CD3+CD8+ T cells increased after treatment with astragaloside IV (**Figure 6C**). This showed that astragaloside IV can promote the activation of T cells and enhance the effect of anti-tumor immunotherapy. All the above experiments showed that GBP1 combined with IDO1 to increase the secretion of IDO1 and promotes the malignant progression of lung cancer. And astragaloside IV can assist the anti-tumor effect of PD-1 by blocking the binding of GBP1 and IDO1 (**Figure 6D**). These results indicate that astragaloside IV can block the combination of IDO1 and GBP1 and reduce the secretion of IDO1, and further enhance the anti-tumor effect of PD-1 inhibitors by reducing T cell exhaustion.

## Discussion

The combination of surgical resection and chemotherapy improves the prognosis of patients with lung cancer. However, additional chemotherapy and targeted therapies remain ineffective in improving the prognosis of patients with metastasis (5, 13). Although the application of immune checkpoint inhibitors has brought hope for cancer immunotherapy, many patients still do not respond to the therapy (14, 15). This phenomenon may be related to the expression of PD-L1 in tumor cells and the tumor microenvironment. Previous study found that inhibiting IDO1 can restore the activity of T cells and improve the ability to kill tumor cells (16–18). Hence, a variety of drug candidates have been developed and are in clinical trials. Most laboratories researching IDO1 agree that it is a cytoplasmic protein. Only Rita Romani’s research has shown that human amniotic fluid stem cells exert immunoregulatory functions through secreted IDO1 (19). However, the role of extracellular secretion of IDO1 in cancer progression has not yet been described. The present study showed that IDO1 can bind to GBP1 and then be secreted outside the cell with the assistance of GBP1. This phenomenon may explain why the high expression of IDO1 alone is not significantly related to the prognosis of patients with lung adenocarcinoma.

GBP1, as a member of the GTPase superfamily, participates in membrane, cytoskeleton and cell verification reactions (20–22). However, in tumors, GBP1 is a double-edged sword. High GBP1 expression has been previously related to better or worse prognosis in different human tumors. In colorectal cancer, GBP1 can inhibit tumor cell proliferation (23). However, in oral squamous cell carcinoma and ovarian cancer, GBP1 confers tumor cells with stronger drug resistance, and its expression level is related to the poor prognosis of patients (24–26). The expression and activity of GBP1 are closely related to the tumor microenvironment (27). Stimulation of inflammatory factors can induce the expression of GBP1, 2 and 5, but only GBP1 is secreted outside the cell (25, 28). In the present study, GBP1 can bind IDO1, thereby transporting IDO1 to the extracellular matrix during the secretion of GBP1. This result suggests that IDO1 only responds to IDO1 inhibitors after it is secreted into the cell matrix through a similar pathway. However, how GBP1 is secreted out of cells in the body and its relationship with tumor microenvironment and inflammation warrant further verification.

The recognition of tumor cells by T cells must be enhanced and T cells must have strong killing ability to promote the clinical application of immune checkpoint inhibitors. If IDO1 is secreted into the extracellular matrix with the assistance of GBP1, it is bound to have a certain inhibitory effect on the activity of T cells (29, 30). In animal models of glioma, the combination of IDO1 and PD-1 inhibitors prolongs the survival time of animals (31, 32). These seem to indicate that inhibiting the activity of IDO1 can enhance the anti-tumor effect of PD-1 inhibitors. In the present study, we used interface inhibitors to break the combination of GBP1 and IDO1, avoid the extracellular localization of IDO1 and solve the root cause of IDO1’s inhibitory effect on T cell activity. Astragaloside IV can block the combination of IDO1 and GBP1 to prevent the exhaustion of T cells caused by the extracellular secretion of IDO1, and further enhance the inhibitory effect of PD-1 inhibitors in lung cancer. Studies have shown that Astragaloside IV has anti-inflammatory and chemotherapy sensitization effects (33–35). Here we found the synergistic anti-tumor effect of astragaloside IV combined with PD-1. Of course, other effects of astragaloside IV may play a certain role in tumor growth in this experiment. However, we did detect that astragaloside IV can block the combination of IDO1 and GBP1. In addition, studies have suggested that astragaloside IV can inhibit the expression of IDO1, which may be inconsistent with our conclusions (36). Our results prove that blocking the extracellular secretion of IDO1 can enhance the therapeutic effect of PD-1.

The interaction of GBP1 and IDO1 induced the extracellular secretion of IDO1 promote lung cancer cell progression. In addition, astragaloside IV can block the interaction between GBP1 and IDO1 and enhance the inhibitory effect of PD-1 inhibitors of lung cancer cell *in vivo* and *in vitro*. These results explain the mechanism of IDO1 in regulating T cell infiltration in lung cancer and provide a certain possibility for its clinical combination therapy with PD-1 inhibitors.

## Data Availability Statement

The original contributions presented in the study are included in the article/**Supplementary Material**; further inquiries can be directed to the corresponding author.

## Ethics Statement

The animal study was reviewed and approved by Affiliated Taizhou hospital of Wenzhou Medical University.

## Author Contributions

HY conceived and designed the study. YM performed the cell and animal experiments and wrote the paper. WW and KC performed cell and molecular experiments. XX performed the pathological analysis. SZ analyzed data. All authors contributed to the article and approved the submitted version.

## Funding

This work was funded by the National Natural Science Foundation of China (81874221) and the Taizhou Science and Technology Bureau (20ywa09).

## Conflict of Interest

The authors declare that the research was conducted in the absence of any commercial or financial relationships that could be construed as a potential conflict of interest.

## Supplementary Material

Supplementary Figure 1 IDO1 binds GBP1 in A549 cells. **(A)** Identification of IDO1 binding protein by silver staining. **(B)** Use Co-IP to detect the interaction between IDO1 and GBP1. **(C)** GST pull-down was performed to analyze the interaction between IDO1 and GBP1 *in vitro*. D. Immunofluorescence confirms the co-localization of IDO1 and GBP1 in A549 cells.
