# Supplementary figures and images for: GBP1 Facilitates Indoleamine 2,3-Dioxygenase Extracellular Secretion to Promote the Malignant Progression of Lung Cancer
Source: Front Immunol. 2021 Jan 20;11:622467. doi: 10.3389/fimmu.2020.622467 (PMC7857027; doi:10.3389/fimmu.2020.622467)

**B**

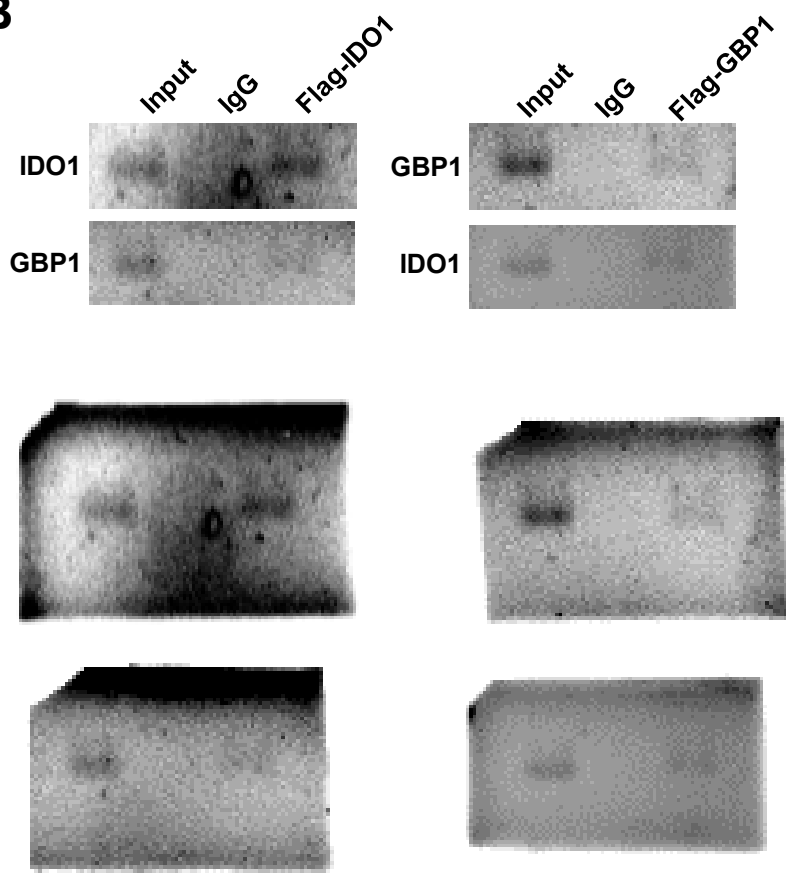

**C**

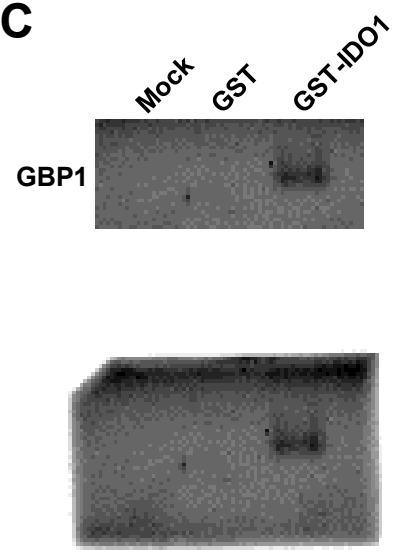

**Figure 1**

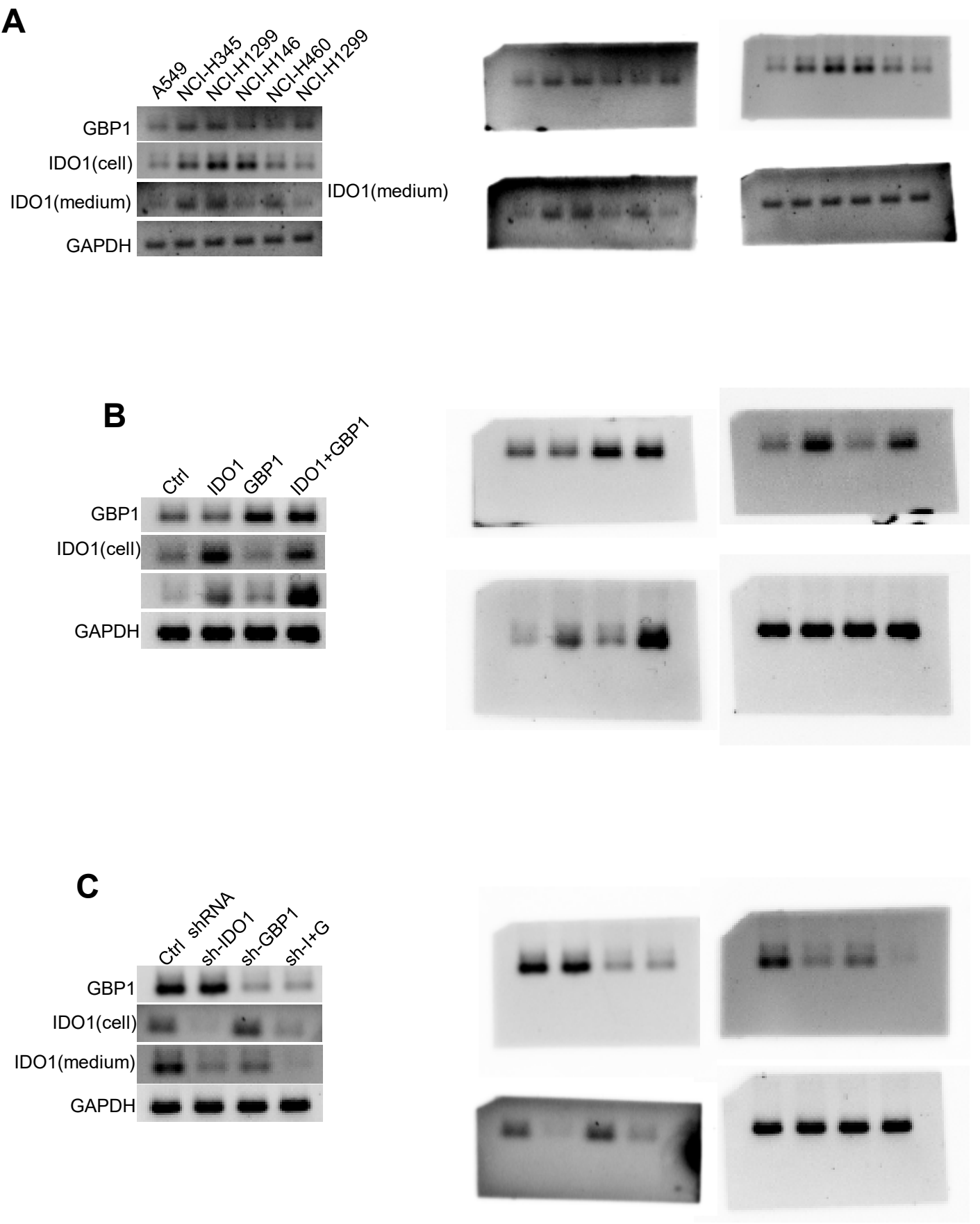

**Figure 2**

B

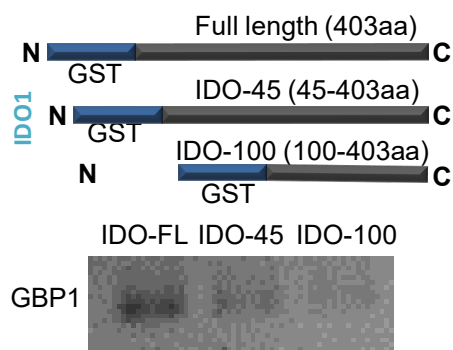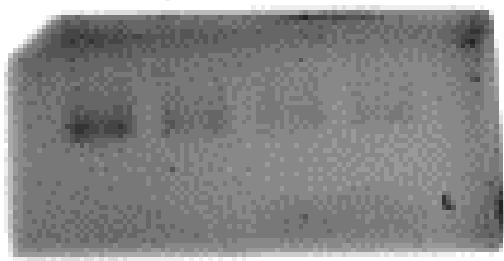

C

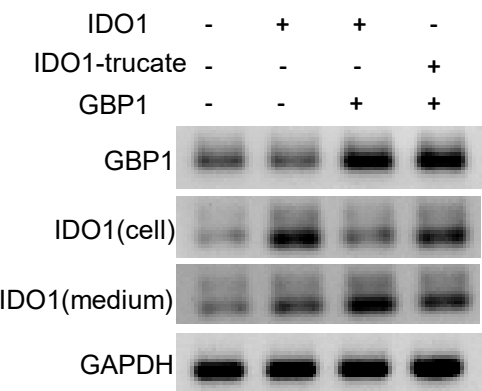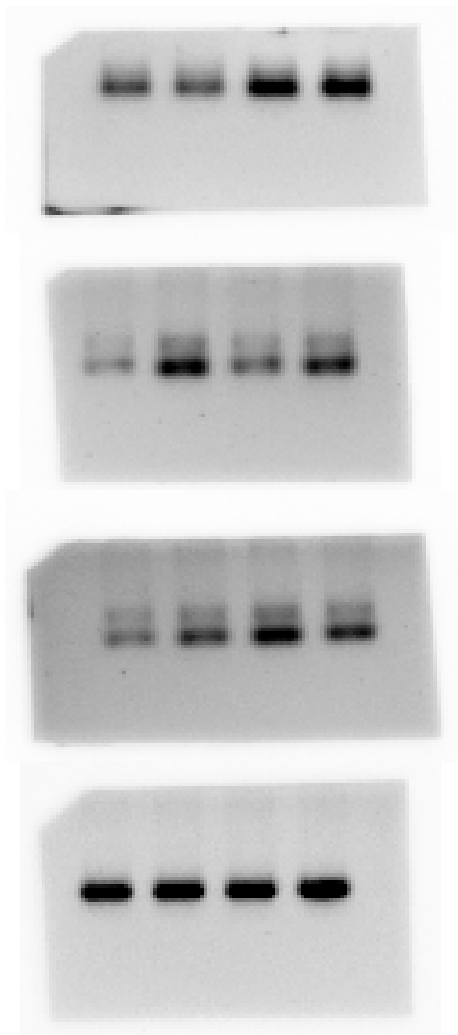

Figure 3

**B**

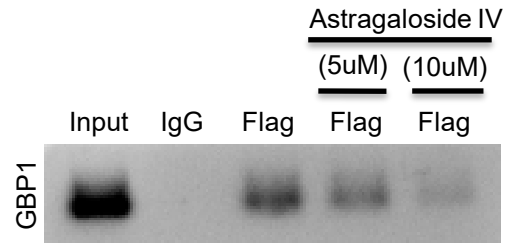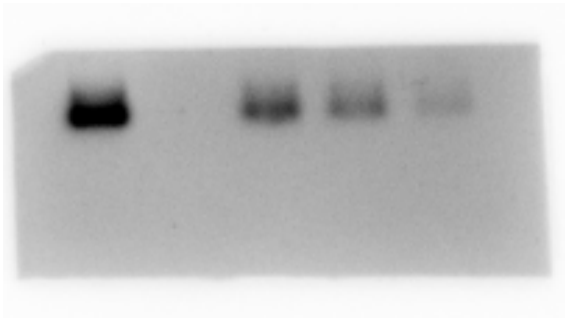

**C**

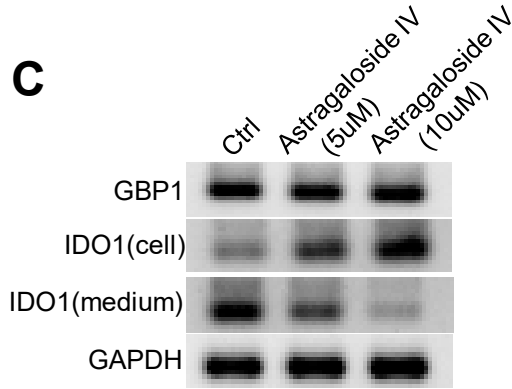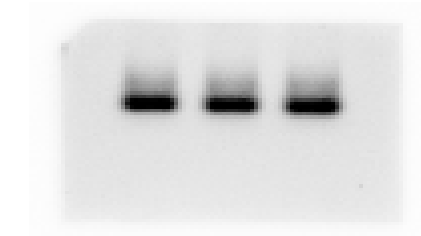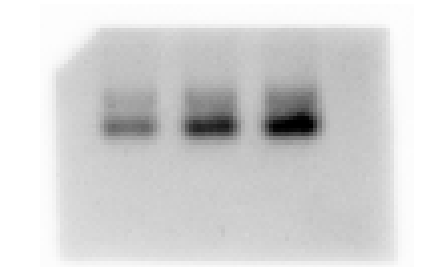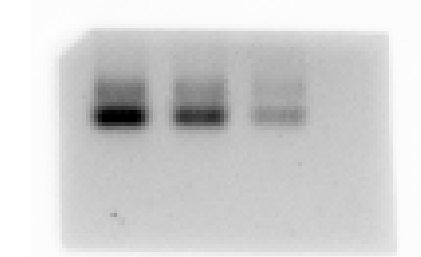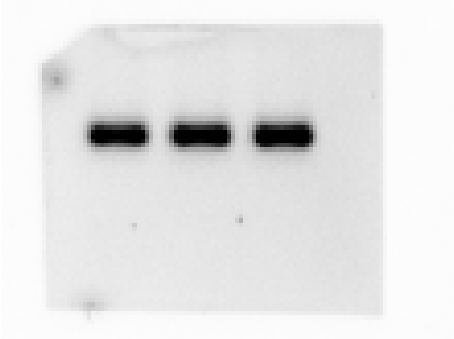

**Figure 5**

Supplement: Supplementary Figure 1 — IDO1 binds GBP1 in A549 cells. (A) Identification of IDO1 binding protein by silver staining. (B) Use Co-IP to detect the interaction between IDO1 and GBP1. (C) GST pull-down was performed to analyze the interaction between IDO1 and GBP1 in vitro. D. Immunofluorescence confirms the co-localization of IDO1 and GBP1 in A549 cells. [file DataSheet_1.pdf]
